# Ablation of the Sam68 RNA Binding Protein Protects Mice from Age-Related Bone Loss

**DOI:** 10.1371/journal.pgen.0010074

**Published:** 2005-12-16

**Authors:** Stéphane Richard, Nazi Torabi, Gladys Valverde Franco, Guy A Tremblay, Taiping Chen, Gillian Vogel, Mélanie Morel, Patrick Cléroux, Alexandre Forget-Richard, Svetlana Komarova, Michel L Tremblay, Wei Li, Ailian Li, Yun Jing Gao, Janet E Henderson

**Affiliations:** 1 Terry Fox Molecular Oncology Group and the Bloomfield Center for Research on Aging, Lady Davis Institute for Medical Research, Sir Mortimer B. Davis Jewish General Hospital, and Departments of Medicine and Oncology, McGill University, Montréal, Québec, Canada; 2 Department of Medicine and Centre for Bone and Periodontal Research, McGill University, Montréal, Québec, Canada; 3 Faculty of Dentistry, McGill University, Montréal, Québec, Canada; 4 McGill Cancer Centre and Department of Biochemistry, McGill University, Montréal, Québec, Canada; Stanford University School of Medicine, United States of America

## Abstract

The Src substrate associated in mitosis of 68 kDa (Sam68) is a KH-type RNA binding protein that has been shown to regulate several aspects of RNA metabolism; however, its physiologic role has remained elusive. Herein we report the generation of Sam68-null mice by homologous recombination. Aged Sam68^−/−^ mice preserved their bone mass, in sharp contrast with 12-month-old wild-type littermates in which bone mass was decreased up to approximately 75%. In fact, the bone volume of the 12-month-old Sam68^−/−^ mice was virtually indistinguishable from that of 4-month-old wild-type or Sam68^−/−^ mice. Sam68^−/−^ bone marrow stromal cells had a differentiation advantage for the osteogenic pathway. Moreover, the knockdown of Sam68 using short hairpin RNA in the embryonic mesenchymal multipotential progenitor C3H10T1/2 cells resulted in more pronounced expression of the mature osteoblast marker osteocalcin when differentiation was induced with bone morphogenetic protein-2. Cultures of mouse embryo fibroblasts generated from Sam68^+/+^ and Sam68^−/−^ littermates were induced to differentiate into adipocytes with culture medium containing pioglitazone and the Sam68^−/−^ mouse embryo fibroblasts shown to have impaired adipocyte differentiation. Furthermore, in vivo it was shown that sections of bone from 12-month-old Sam68^−/−^ mice had few marrow adipocytes compared with their age-matched wild-type littermate controls, which exhibited fatty bone marrow. Our findings identify endogenous Sam68 as a positive regulator of adipocyte differentiation and a negative regulator of osteoblast differentiation, which is consistent with Sam68 being a modulator of bone marrow mesenchymal cell differentiation, and hence bone metabolism, in aged mice.

## Synopsis

Osteoporosis is a debilitating bone disease that is characterized by reduced bone mass and microarchitectural damage, which result in increased bone fragility and susceptibility to fracture. Peak bone mass, which is achieved by the age of 30 in humans, has been identified as a major determinant of resistance or susceptibility to osteoporosis. The authors generated mice deficient for the Sam68 RNA binding protein, a protein of unknown physiologic function. The mice develop normally and are protected against bone loss during aging. Age-related bone loss has long been associated with an increase in marrow adipocytes, which are derived from the same mesenchymal lineage as osteoblasts in bone marrow. The authors showed that Sam68 regulates the differentiation of this mesenchymal lineage, such that in its absence, osteoblasts continued to be generated in aging bone, leading to preservation of bone mass. This study identifies a physiologic role for Sam68 as a modulator of the bone marrow stem cell niche and hence of bone metabolism. The data identify Sam68 as a potential therapeutic target for the prevention and treatment of age-related bone loss.

## Introduction

During skeletal development, the anabolic activity of osteoblasts [1] is favored over the catabolic activity of osteoclasts [2], which results in a net gain in bone mass. At skeletal maturity, bone mass is maintained through the balanced activity of osteoblasts and osteoclasts during the remodeling cycle. During skeletal aging, there is a shift in the balance that favors osteoclast over osteoblast activity, which results in net bone loss [3]. The amount and rate at which bone is gained during development and lost during aging are determined in large part by genetics [4–6] but also by physical activity and by alterations in the availability and response of bone cells to circulating hormones [7–9] and locally derived growth factors [10,11]. Whereas genetic-based studies have provided novel insights into the pathways that regulate bone development [12–14], relatively little is known about the etiology of age-related bone loss. Increased bone resorption in elderly men and women is associated with a reduction in bone mass and an increase in circulating levels of bone biomarkers [15]. These changes have been attributed primarily to nutritional deficits resulting in alterations in the parathyroid hormone–vitamin D axis [16], to gonadal hormone deficiency [17], to leptin levels and the sympathetic nervous system [8,18–20], and to alterations in bone cell apoptosis [21]. Bone loss in the elderly has also been attributed to alterations in the response of bone marrow stromal cells to their microenvironment that favors differentiation down the adipocyte lineage rather than the osteoblast lineage [22].

Aging has long been associated with an increase in marrow fat, where the generation of adipocytes is favored over osteoblasts [23]. Osteoblasts and adipocytes are derived from a common mesenchymal precursor cell present in bone marrow, and the factors that control this age-induced switch toward adipogenic differentiation is not well understood [24]. While several transcriptional regulatory proteins have been associated with cell fate determination of bone marrow mesenchymal cells, including peroxisome proliferator–activated receptor γ (PPARγ) and KLF5 [25–30], the role of RNA binding proteins in this process remains unknown. RNA binding proteins of the KH type are known regulators of cellular differentiation. For example, expression defects in the KH domain proteins NOVA and FMRP are known to cause paraneoplastic neurologic disorders [31] and the fragile X syndrome, respectively, in humans [32]. The phenotype of the *quaking viable* mice suggests a role for the QUAKING RNA binding protein in oligodendrocytes and myelination [33]. Indeed, the ectopic expression of the QKI-6/7 isoforms in vivo led to the formation of glial cells rather than neurons from neural progenitor, demonstrating its role in cell fate determination [34]. The loss of GLD-1 protein in *Caenorhabditis elegans* prevents the appearance of stem cells [35], and the absence of the KH domain protein HOW in *Drosophila* prevents muscle differentiation and results in flies with held-out-wings [36,37]. The Src substrate associated in mitosis of 68 kDa (Sam68) is also a member of the family of KH domain RNA binding proteins [38]; however, its physiologic role has remained undefined.

Sam68 was identified as an SH3 and SH2 domain interacting protein for Src family kinases and is also a known substrate of Src kinases [39–42] and of the breast tumor kinase [43]. Sam68 has been shown to facilitate the export of unspliced HIV RNA [44] and to regulate pre-mRNA processing [45]. In the present paper, we report the generation of Sam68^−/−^ mice and analysis of their skeletal phenotype. Our data indicate that the absence of Sam68 confers resistance to age-related bone loss in mice such that old Sam68 have a higher bone mass than their wild-type littermates. We provide evidence that Sam68 regulates the differentiation of bone marrow stromal cells by showing that cells isolated from Sam68^−/−^ animals had enhanced osteogenic activity and decreased adipogenic activity than those harvested from wild-type littermates. Furthermore, Sam68^−/−^ mouse embryo fibroblasts (MEFs) were impaired in their capacity to differentiate into adipocytes, consistent with Sam68 being a regulator of bone marrow mesenchymal cell differentiation. These results also characterize a new animal model to study bone metabolism, regeneration, and repair during aging.

## Results

### Sam68 Is Expressed in the Developing Skeleton of Embryonic Mice

**Figure 1:**
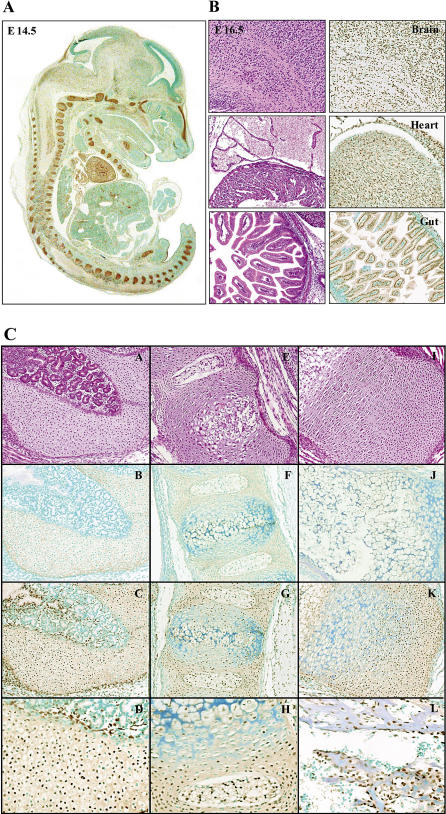
Immunohistochemical Localization of Sam68 in Embryonic Mice (A) Embryonic mice were removed from pregnant dams at E14.5 and E16.5, fixed in 4% paraformaldehyde, and embedded in paraffin. The entire embryo was immunostained with the AD1 anti-Sam68 antibody and counterstained with methyl green, and the image was captured at ×1.2 magnification. (B) Embryonic soft tissues from the brain, heart and gut were stained with hematoxylin (left) and immunostained with anti-Sam68 antibody (right), and images were captured at ×20 magnification. (C) Intense anti-Sam68 immunoreactivity was seen in chondrocytes in the nasal septum (panels A–D), in developing vertebra (panels E–H), and in the femoral epiphysis (panels I–K), as well as in diaphyseal osteoblasts (panel L). Adjacent sections were stained with hematoxylin and eosin (panels A, E, and I) or with antibody preadsorbed with the immunizing peptide (panels B, F, and J). Sam68 was localized primarily in the nucleus of cells in a variety of tissues but was also found occasionally in the cytoplasm. Magnification at source ×20, except for panels D, H, and L, which were ×40. Staining patterns are representative of three to five embryos.

The Sam68 mRNA is known to be widely expressed [38], whereas its pattern of protein expression in vivo remains to be defined. Sections of paraffin-embedded wild-type E14.5 and E16.5 embryonic mice were immunostained with the well-characterized AD1 anti-Sam68 antibody, raised in rabbits against an immunizing peptide that corresponds to amino acids 330 to 348 of the mouse Sam68 protein [46]. Sam68 immunoreactivity was observed in the skeleton and soft tissues of developing wild-type mice at E14.5 and E16.5 (Figure 1). Intense staining was seen in the nucleus of cells in the developing brain, heart, and small intestine (Figure 1B), as well as in chondrocytes in the nasal septum and the glandular tissue adjacent to the nasal cartilage (Figure 1C, panels A–D), in the vertebra and intervertebral discs (panels E–H) and in the epiphysis (panels I–K) and metaphysis (panel L) of long bones. Nuclear staining was observed in proliferating chondrocytes and hypertrophic chondrocytes (Figure 1C, panel H), in osteoblasts (panel L; Dataset S1), and in osteoclasts (Dataset S1). No staining was observed with preimmune serum or when the antiserum was preadsorbed with the immunizing peptide (Figure 1C, panels B, F, and J). Taken together, these data demonstrate that Sam68 protein is selectively expressed in the developing mouse embryo, with particularly elevated expression in cartilage and bone.

### Sam68 is Not Essential for Mouse Development

**Figure 2:**
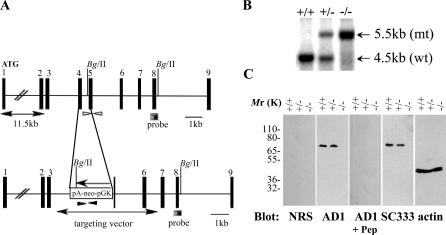
Generation of Sam68-Deficient Mice (A) The genomic organizations of the wild-type and targeted *sam68* alleles after homologous recombination are depicted. The location of the DNA fragment used as a probe for the Southern blot analysis is shown, as well as the sizes of the two BglII fragments detected for wild-type and targeted *sam68* alleles. The targeted allele replaces exon 4 and part of exon 5 of *sam68* with a PGK-neomycin cassette. (B) Southern-blot analysis of genomic DNA from wild-type (+/+), heterozygous (+/−), and homozygous (−/−) mice. DNA fragments corresponding to wild-type (4.5 kb) and the targeted (5.5 kb) alleles are illustrated. (C) Western blot analysis of Sam68 expression. Protein extracts from wild-type, heterozygous, and homozygous cells subjected to immunoblot analyses using normal rabbit serum, anti-Sam68 AD1 antibody, the peptide antibody AD1 preabsorbed with the immunogenic peptide corresponding to amino acids 330–348 of mouse Sam68, anti-Sam68 Sc333 antibody that recognizes the C-terminal 20 amino acids of Sam68, and anti-actin antibodies as loading control. The migration of the molecular mass markers is known on the left in kDa.

To define the physiologic role of Sam68, we generated Sam68-deficient mice by targeted disruption of exons 4 and 5 of the *sam68* gene, which encode the functional region of the KH-type RNA binding domain (Figure 2A). The integrity of the targeted allele was verified by Southern blot analysis (Figure 2B) and by PCR of genomic DNA (unpublished data). Sam68 transcripts encoded by exons 1 to 5 were absent as evidenced by RT-PCR (Dataset S2), and Sam68^−/−^ mice were devoid of Sam68 protein, as visualized by immunoblotting with anti-Sam68 AD1 and SC-333 antibodies or control normal rabbit serum or anti-Sam68 AD1 preabsorbed with peptide (Figure 2C). SC-333 is a rabbit anti-Sam68 antibody that was raised against the C-terminal 20 amino acids of Sam68 [47]. These data confirmed the generation of a mouse deficient in Sam68. The genotypes of offspring from heterozygote intercrosses exhibited a Mendelian segregation at E18.5 (Table 1). Despite the lack of visible deformity, many of the Sam68^−/−^ pups died at birth of unknown causes. Sam68^+/-^ mice were phenotypically normal and Sam68^−/−^ pups that survived the perinatal period invariably lived to old age. Despite evidence that Sam68 mRNA is widely expressed and phosphorylated in mitosis [41,42], the Sam68^−/−^ mice did not develop tumors and showed no immunologic or other major illnesses. Sam68^−/−^ mice did, however, have difficulty breeding due to male infertility and the females rarely provided adequate care to their young.

**Table 1:**
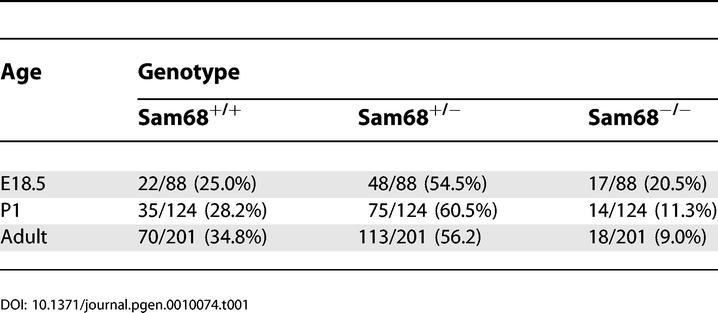
Progeny of Sam68 Heterozygote Breeding

### Sam68 Deficiency Protects Mice from Age-Related Bone Loss

**Figure 3:**
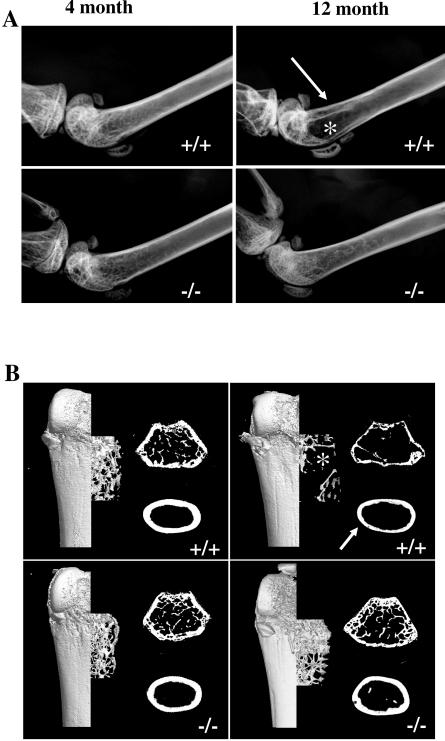
Radiologic Assessment of the Femur of Young and Old Sam68^+/+^ and Sam68^−/−^ Mice (A) Mice were given a lethal dose of anesthetic at the indicated times, and contact radiographs of the distal femora were obtained on a Faxitron MX20 equipped with an FPX-2 Imaging system. Representative radiographs of the distal femur of Sam68^+/+^ (+/+) and Sam68^−/−^ (−/−) mice revealed comparable radiopacity at 4 months (left). At 12 months (right), cortical thinning (arrow) and radiolucency (asterisk) were apparent in the distal femur of +/+ mice but not −/− mice. (B) Bones were dissected free of soft tissue and fixed overnight in 4% paraformaldehyde before scanning on a Skyscan 1072 static instrument equipped with 3D Creator analytical software. Representative three-dimensional re-constructions and two-dimensional cross-sectional scans demonstrated similar architecture in the distal femur of Sam68^+/+^ (+/+) and Sam68^−/−^ (−/−) mice. In keeping with the results from Faxitron x-ray, trabecular bone (asterisk) and cortical thickness (arrow) were reduced in the femur of 12-month-old +/+ mice compared with all other groups**.** The images are representative of those from five to seven animals in each group.

Src null animals are known to have bone metabolism defects [48], and since Sam68 is a substrate of Src, we decided to analyze the Sam68 mice for skeletal abnormalities. Cohorts of Sam68^+/+^ and Sam68^−/−^ mice were euthanized at 4 and 12 months of age for skeletal phenotyping. To minimize differences in the bone phenotype that might arise secondary to gender or weight differences, we selected age-matched female mice for these analyses. The female mice demonstrated similar increases in body weight, although 4-month-old Sam68^−/−^ mice weighed less than Sam68^+/+^ mice, and similar changes in bone lengths in the axial and appendicular skeleton between 4 and 12 months of age (Table 2). Faxitron radiography (Faxitron X-ray Corporation, Wheeling, Illinois, United States) (Figure 3A) and micro–computed tomography (CT) (Figure 3B) revealed significant cortical thinning (arrow) and a reduction in metaphyseal bone (asterisk) in the distal femora of 12-month-old Sam68^+/+^ mice compared with 4-month-old mice of either genotype and 12-month-old Sam68^−/−^ mice. Similar reductions in trabecular bone were shown by Faxitron and micro-CT in the fifth lumbar vertebrae of the 12-month-old Sam68^+/+^ mice but not in the Sam68^−/−^ mice (unpublished data). Total body bone mineral content (BMC), quantified with a Lunar PIXImus mouse densitometer (GE-Lunar, Madison, Wisconsin, United States), increased in both Sam68^+/+^ and Sam68^−/−^ mice between 4 months and 12 months of age, but only reached significance in the Sam68^−/−^ mice (Table 2). Similarly, a greater increase in BMC was seen in the femur and vertebra in the Sam68^−/−^ mice. Bone mineral density (BMD) remained constant or decreased in the wild type mice but increased significantly in the total body and in the femoral and vertebral regions of interest in the Sam68^−/−^ (Table 2). These data showed that Sam68^−/−^ mice continued to thrive and accrue bone in the axial and appendicular skeleton for longer than 12 months. This was in contrast to the situation in age-matched, littermate controls in which a significant amount of bone was lost over the same timeframe.

**Table 2:**
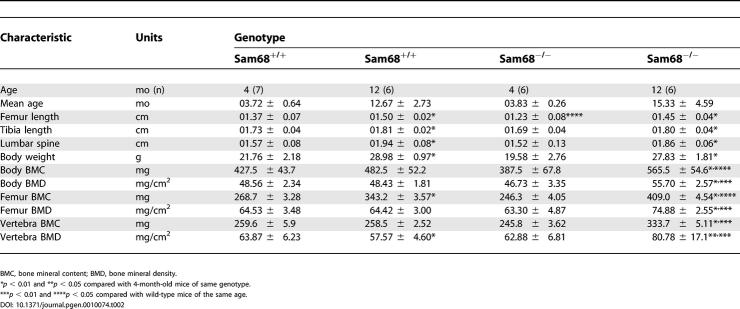
Morphology and Bone Mineral Density in 4- and 12-Month-Old Female Mice

### Three-Dimensional Architecture of Bone Is Preserved in Aged Sam68^−/−^ Mice

**Figure 4:**
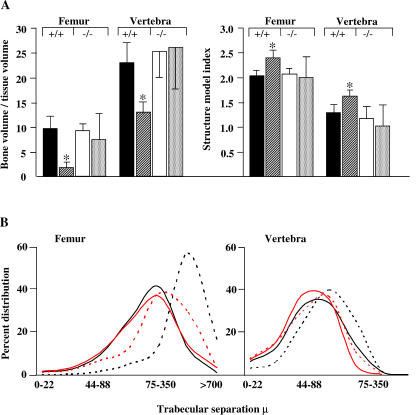
Quantitative Micro-CT of Trabecular Bone Composition and Architecture (A) Bone volume/tissue volume (BV/TV) and structure model index (SMI) were calculated on the femur and fourth lumbar vertebra of six or seven mice in each group using 3D Creator software supplied with the Skyscan instrument. Results expressed as the mean ± SD showed significant differences (*p* < 0.01) between 4-month-old Sam68^+/+^ mice (solid black) and 12-month-old Sam68^+/+^ mice (hatched black) but not between 4-month-old Sam68^−/−^ mice (solid white) and 12-month-old Sam68^−/−^ mice (stippled white). (B) The distance between trabeculae was reflected in a shift to the right of the distribution curves for 12-month-old Sam68^+/+^. Solid black = 4-month-old Sam68^+/+^; hatched black = 12-month-old Sam68^+/+^; solid red = 4-month-old Sam68^−/−^; stippled red = 12-month-old Sam68^−/−^. The asterisks denote *p* < 0.01.

Bone loss and compromised architecture are characteristic features of the skeletons of aged C57BL/6 mice and resemble the clinical features of age-related bone loss in humans that can predispose an individual to fracture. Quantification of the micro-CT data shown in Figure 3B confirmed the reduction in bone volume compared with tissue volume (BV/TV; Figure 4A, left) in the 12-month-old Sam68^+/+^ mice (hatched bars) compared with 4-month-old mice of both genotypes (solid bars) and 12-month-old Sam68^−/−^ mice (stippled bars). This was associated with a significant increase (*p* < 0.01; denoted by the asterisks) in the structure model index, which measures the ratio of plate-like to rod-like structures (Figure 4A, right). A quantifiable increase in the mean trabecular separation (Tb.Sp) (unpublished data) in 12-month-old Sam68^+/+^ mice was due to an increase in the percentage of spaces falling in the range of 350 to 700 μm (Figure 4B, red hatched line). Trabecular thickness remained constant among the different groups of mice (unpublished data). In effect, this meant that there were fewer trabeculae, rather than equivalent numbers of thin trabeculae, in the 12-month-old Sam68^+/+^ mice compared with any of the other groups of mice.

### Bone Remodeling Is Preserved in Aged Sam68^−/−^ Mice

**Figure 5:**
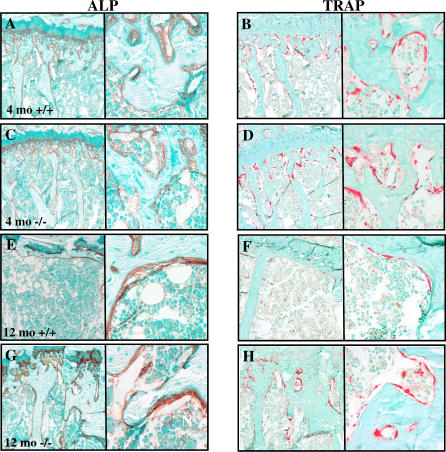
Histologic Analysis of Undecalcified Bone from Sam68**^+/+^** and Sam68^−/−^ Mice Sections of tibia fixed in 4% paraformaldehyde and embedded in plastic were stained for ALP (A–C, E–G) activity to identify osteoblasts or for TRAP (B–D, F–H) activity to identify osteoclasts. Staining patterns were similar in 4-month-old Sam68^+/+^ (A and B), 4-month-old Sam68^−/−^ (C and D), and 12-month-old Sam68^−/−^ (G and H) mice compared with 12-month-old Sam68^+/+^ mice (E and F). Magnification at source, left panels ×10 and right panels ×40. Micrographs are representative of those taken from five to seven sections in each group of animals.

To further define the mechanisms involved in the preservation of bone mass in aged Sam68^−/−^ mice, we prepared sections from plastic-embedded femora and tibia to evaluate osteoblast and osteoclast activity (Figure 5). In situ enzyme histochemical staining for alkaline phosphatase (ALP; brown stain) activity was used as a biomarker for osteoblasts and tartrate-resistant ALP (tartrate-resistant acid phosphatase [TRAP]; red stain) activity as a marker for osteoclasts. Little difference was seen between Sam68^+/+^ (Figure 5A and 5B) and Sam68^−/−^ (Figure 5C and 5D) mice at 4 months of age. ALP- and TRAP-positive cells were reduced in the 12-month-old Sam68^+/+^ mice (Figure 5E and 5F) and remained unchanged in 12-month-old Sam68^−/−^ mice (Figure 5G and 5H). The reduction in both osteoblast and osteoclast activity in the 12-month-old Sam68^+/+^ mice argued against bone being lost primarily due to a relative increase in osteoclast over osteoblast activity, as seen in high turnover disease [49].

Histomorphometric analyses [50] of the long bones (Table 3) corroborated the radiologic evidence of age-related bone loss. Bone volume (BV/TV), newly formed osteoid (OV/TV), and mineral apposition rate (MAR) were all significantly reduced in 12-month-old Sam68^+/+^ mice compared with 4-month-old Sam68^+/+^ mice. These reductions were associated with a significant increase in marrow fat (FV/TV) and decreased numbers of osteoblasts (nOB/TV) and osteoclasts (nOC/TV) per tissue volume (Table 3). These results were in contrast to those of the 12-month-old Sam68^−/−^ mice in which all histomorphometric parameters, including marrow fat, resembled those of young mice of either genotype (Table 3). When cells were expressed as a function of the bone perimeter (nOB/BP, nOC/BP), there were no statistical differences and the ratio of osteoblasts to osteoclasts was similar in all groups of mice (Table 3).

**Table 3:**
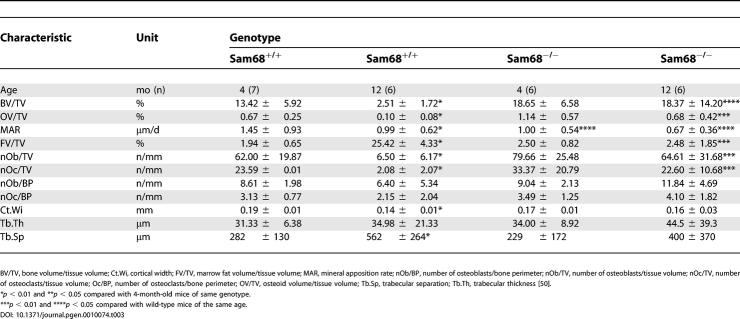
Histomorphometric Analysis of Long Bones from 4- and 12-Month-Old Mice

Circulating levels of serum C-telopeptide (sCTX; Roche Diagnostics, Mannheim, Germany) and ALP showed little difference among the groups, except for a small decrease in 12-month-old Sam68^−/−^ mice and (Dataset S3). Serum estrogen was significantly decreased in all 12-month-old mice regardless of genotype with a reciprocal increase in interleukin-6 levels (Dataset S3). Given the involvement of leptin in regulating body and bone mass, serum leptin was measured in Sam68^+/+^ and Sam68^−/−^ mice. Twelve-month-old Sam68^−/−^ mice had significantly lower levels than young and old Sam68^+/+^ mice and young Sam68^−/−^ mice (Dataset S3). Taken together, these observations suggested that preservation of bone mass in the Sam68^−/−^ mice was not due primarily to altered estrogen status but could have been influenced by differences in circulating leptin levels.

### Osteoblast, but Not Osteoclast, Activity Is Altered in Sam68^−/−^ Mice Ex Vivo

**Figure 6:**
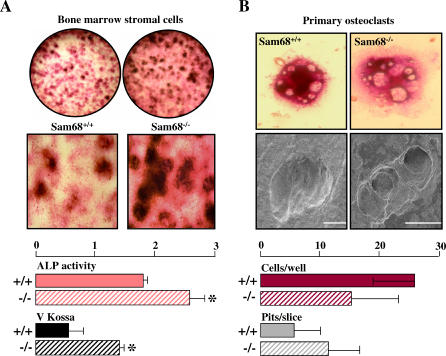
Ex Vivo Activity of Sam68^+/+^ and Sam68^−/−^ Osteoblasts and Osteoclasts Marrow stromal cells were isolated from the long bones of juvenile mice and maintained under conditions that promote osteoblast differentiation. (A) Cultures were fixed in 4% paraformaldehyde after 6 or 18 days and stained in situ for ALP activity and with silver nitrate (von Kossa) to detect mineralized nodules. Sam68^−/−^ cultures stained more intensely for ALP at early and late time points and produced significantly more mineralized nodules after 18 days. Asterisks represent *p* < 0.01. (B) Primary osteoclasts were isolated from the crushed long bones of the same mice and plated on glass coverslips or on dentin slices to quantify numbers and activity, respectively. Osteoclasts were identified as cells with three or more nuclei that stained positive for TRAP activity (upper) and excavated pits in dentin slices, as demonstrated by SEM (lower, bar = 20 μm). No statistical differences were observed either in the number of TRAP-positive cells or in their resorptive activity.

Maintenance of bone mass during the adult remodeling cycle is dependent on the coupling of osteoblast to osteoclast activity, such that there is no net gain or loss of bone [49]. To further explore the age-related advantage of Sam68^−/−^ mice with respect to bone preservation, we examined the functional activity of Sam68^−/−^ osteoblasts and osteoclasts ex vivo (Figure 6A and 6B). Cultures of bone marrow stromal cells harvested from 4-week-old Sam68^−/−^ mice and maintained for 18 days in osteoblast differentiation medium demonstrated more intense staining for ALP at 6 days (unpublished data) and 18 days (Figure 6A), and more mineralized nodules at 18 days, than the Sam68^+/+^ mice, even though similar amounts of fibroblast colony-forming units (CFU-F) were observed (Figure 6A and unpublished data). RT-PCR analysis of molecular markers of osteoblast differentiation (Dataset S2) revealed similar increases over time in RNA from Sam68^+/+^ and Sam68^−/−^ mice, although type I collagen appeared to be up-regulated at both timepoints in the Sam68^−/−^ mice. Short-term cultures of mature osteoclasts released from the crushed long bones of Sam68^+/+^ and Sam68^−/−^ mice stained equally well for TRAP and excavated approximately equal numbers of pits of equal size in dentin slices, as visualized by scanning electron microscopy (Figure 6B). These findings suggest that the osteoclasts may not be the primary defect in Sam68^−/−^ mice, as they are in Src null mice [48].

### The Absence of Sam68 Prevents Adipocyte Differentiation and Promotes Osteoblast Differentiation

**Figure 7:**
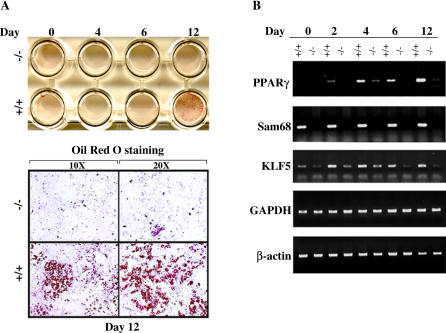
Ex Vivo Adipogenesis Analysis of Sam68^−/−^ Mouse Embryonic Fibroblasts MEFs were isolated from mouse embryos at embryonic day 14.5. Equal number of MEFs from Sam68^+/+^ and Sam68^−/−^ was plated on glass cover slips in 24 well-plates. Adipocyte differentiation was carried out at indicated times by the addition of complete media containing the pioglitazone. (A) Cultures were fixed in 4% paraformaldehyde and stained with Oil Red O to detect the fat droplets stored in adipocytes and photographed (top). The cell images were magnified ×10 and ×20 as indicated. (B) RT-PCR was carried out on total cellular RNA isolated after differentiation of the MEFs for day 0, 2, 4, 6, and 12. The DNA fragments were visualized on agarose gels stained with ethidium bromide. The expression of adipogenic markers C/EBPβ, C/EBPδ, PPARα, and KLF5 was examined as well as the expression of controls including Sam68, β-actin, and GAPDH.

It is well recognized that bone marrow stromal cells give rise to both osteoblasts and adipocytes and that age-related bone loss is accompanied by an increase in differentiation down the adipocyte lineage [22]. Therefore, the loss of Sam68 could influence the bone marrow stromal cells to differentiate along the osteogenic versus the adipogenic pathway. Alternatively, the loss of Sam68 could indirectly regulate bone mass as a general disturbance of neuroendocrine control as was shown when leptin and the sympathetic nervous system axis were shown to negatively regulate bone mass [8]. To confirm a role for Sam68 in the regulation of adipocyte differentiation, we isolated primary MEFs from 14.5-day-old Sam68^+/+^ and Sam68^−/−^ embryos. The primary Sam68^−/−^ MEFs were differentiated into adipocytes in vitro in culture medium containing 5 μM pioglitazone to induce adipogenesis. Cells at days 0, 4, 6, and 12 were stained with Oil red O to monitor adipogenesis. Adipogenesis was more pronounced in the wild-type MEFs cultures than in the Sam68^−/−^ MEFs, consistent with the positive role of endogenous Sam68 in adipocyte differentiation (Figure 7). The expression of key transcription factors including the PPARγ and KLF5 was impaired in Sam68^−/−^ differentiated MEFs compared with Sam68^+/+^ MEFs, consistent with impaired adipogenesis in the absence of Sam68 (Figure 7). These data, together with data confirming a lean phenotype in Sam68^−/−^ mice (N. Torabi and S. Richard, unpublished data), support the hypothesis that Sam68 modulates the differentiation of mesenchymal cells.

**Figure 8:**
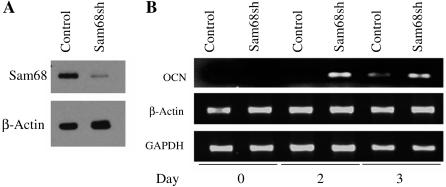
Enhanced Osteogenic Differentiation of the C3HT101/2 Embryonic Cell Line Depleted of Endogenous Sam68 (A) C3HT101/2 cells transfected with an empty vector (pSuper-retro) or a vector containing an shRNA (Sam68 shRNA) were selected with puromycin, and knockdown populations depleted of Sam68 were identified. The reduction in Sam68 protein was analyzed by immunoblotting with anti-Sam68 (AD1) antibody and anti–β-actin antibodies as loading controls. (B) Osteogenic differentiation was carried out with conditioned medium containing BMP-2 for the indicated times. To assess the level of osteogenic differentiation in these cells, expression of late osteoblast marker, osteocalcin (OCN), was analyzed by RT-PCR and compared with β-actin and GAPDH controls. The DNA fragments were visualized by agarose gel stained with ethidium bromide.

To further examine this phenotype in a cell autonomous system, we chose the embryonic mesenchymal multipotential progenitor cells C3H10T1/2. The addition of BMP-2 induced osteoblast differentiation, as evidenced by an approximately 400-fold increase in expression of the osteocalcin *(OCN)* gene [51]. Populations of C3H10T1/2 cells stably transfected with either pSuper-retro (control) and pSuper-retro harboring a short hairpin against Sam68 (Sam68sh) were selected with puromycin. The expression of Sam68 was reduced by approximately 80% as evidenced by immunoblot analyses using β-actin as a loading control (Figure 8A). Osteoblast differentiation was induced in cultures expressing pSuper-retro or pSuper-retro Sam68sh by addition of BMP-2 to the culture medium. . The expression of *OCN* and *β-actin* mRNAs was examined by semiquantitative RT-PCR and the Sam68sh-expressing cells displayed a more pronounced osteoblast phenotype compared with control cells, as assessed by the expression of *OCN* (Figure 8B).

**Figure 9:**
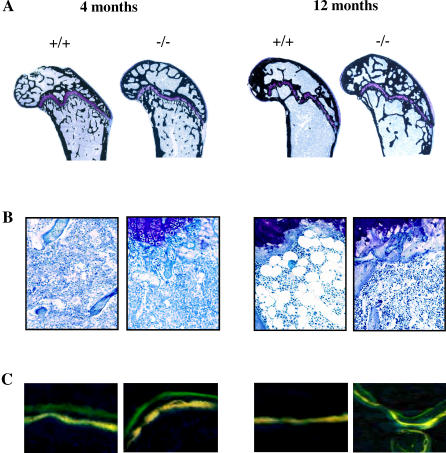
Old Sam68^−/−^ Mice Are Protected from the Development of Fatty Bone Marrow Sections of undecalcified bone were stained with von Kossa and toluidine blue and images captured at original magnifications of ×2 (A), ×40 (B and C) to evaluate mineralized tissue (A, black), marrow adipocytes (B, white), and the mineralization fronts (C, yellow and green). The 12-month-old Sam68^+/+^ bone demonstrated a significant reduction in bone (A) and increase in marrow adipocytes (B) and a decrease in the distance between two consecutive fluorochrome labels (C). Magnification at source was ×40. Micrographs are representative of four to six screened in each group of animals.

Given the apparent enhancement of mineralized nodule formation by Sam68^−/−^ bone marrow stromal cells ex vivo and the phenotype observed with short hairpin RNA (shRNA)-treated C3H10T1/2, we stained sections of bone from 4- and 12-month-old mice for evidence of changes in marrow adiposity. Figure 9 shows sections of undecalcified bone from 4- and 12-month-old Sam68^+/+^ and Sam68^−/−^ mice stained with von Kossa and toluidine blue to show mineralized tissue (Figure 9A, black) and marrow adipocytes (Figure 9B, white) and left unstained to show fluorochrome labeling of the mineralization fronts (Figure 9C). Twelve-month-old Sam68^+/+^ mice showed a noticeable decrease in trabecular bone (Figure 9A), which was associated with a significant increase in marrow adiposity (Figure 9B) and with the two fluorochrome labels superimposed upon one another (Figure 9C). In contrast, the bones of 12-month-old Sam68^−/−^ mice appeared similar to those of the 4-month-old mice of either genotype. These data demonstrate that Sam68 regulates the differentiation of bone marrow mesenchymal cells to promote adipocyte differentiation and inhibit osteoblast differentiation in aging bone.

## Discussion

The present study provides evidence that a physiologic role of Sam68 is to modulate bone marrow mesenchymal stem cells. Young Sam68^−/−^ mice developed normally and contained similar bone mass compared with wild-type littermates. Aged (12 months) Sam68^−/−^ mice displayed a high bone mass phenotype compared with Sam68^+/+^ littermates. The wild-type littermates underwent age-related bone loss that occurs naturally in mammals, while the Sam68^−/−^ animals preserved their bone mass with aging. The differentiation of bone marrow stem cells isolated from Sam68^−/−^ mice and embryonic mesenchymal multipotential progenitor cells C3H10T1/2 treated with Sam68 shRNA resulted in a more pronounced osteoblast differentiation. These findings demonstrate that the loss of Sam68 enhances osteoblast differentiation. The converse was also true, as MEFs isolated from Sam68^−/−^ animals were impaired in their ability to undergo adipocyte differentiation compared with Sam68^+/+^ MEFs. These findings suggest that a physiologic role for Sam68 is to regulate the balance between adipogenic and osteogenic differentiation of the bone marrow mesenchymal.

Sam68 protein expression was observed throughout the developing mouse embryo, in keeping with previous reports that identified the Sam68 mRNA to be widely expressed [38]. We observe Sam68 staining in the brain, heart, and intestine (see Figure 1) as well as liver, skin, and kidney (unpublished data) of late-stage embryos. This expression pattern is consistent with previous work that has observed Sam68 in neurons [52] and as a substrate of the intestinal SIK/breast tumor kinase [43]. The expression of Sam68 was not altered in aging bone marrow stromal cells or in senescencing WI-38 cells (unpublished data). The presence of Sam68 in chondrocytes, osteoblasts and osteoclasts in developing cartilage and bone predicted a pivotal role for the protein in skeletal development.

The Sam68^−/−^ mice were generated using a traditional targeting approach where the functional KH domain was deleted and approximately one third of the Sam68^−/−^ mice survived to adulthood with no apparent defects. One explanation for this phenomenon is that Sam68 function is sub-served by one or more of its family members during development such as SLM-1 and SLM-2 [47]. Alternatively, Sam68 is not required during embryonic development. However, two thirds of the Sam68^−/−^, but not the Sam68^+/-^ pups, were killed by their Sam68^+/-^ mothers. These findings suggest that the mothers are able to detect a subtle defect/difference that we cannot. Adult Sam68^−/−^ mice live a normal life span of approximately two years. The Sam68^−/−^ males were sterile and the Sam68^−/−^ females provided inadequate care to their young, but the pups were not scattered and neglected as observed with FosB^−/−^ mice [53]. Serum levels of estrogen decreased in aging Sam68^−/−^ females as expected; however, the leptin levels decreased in aged Sam68^−/−^ females. The aged Sam68^−/−^ females were not obese and actually weighed less than the littermate controls (see Table 2). Moreover, their appetite was not altered with aging (N. Torabi and S. Richard, unpublished data), suggesting that the observed leptin reduction was not recreating ob/ob-like phenotypes related to weight, appetite and female sterility [54].

The skeletal phenotyping of cohorts of Sam68^+/+^ and Sam68^−/−^ mice showed that bone mass was preserved in aged Sam68^−/−^ mice. Traditional histology and histomorphometry suggested that the mechanism involved preservation of osteoblast and osteoclast activity. The documented role of the Sam68 regulatory protein, Src, in osteopetrosis led us to investigate the morphology and activity of Sam68^−/−^ osteoclasts ex vivo. The Src tyrosine kinase was shown to play a role in bone remodeling when Src^−/−^ mice died at 6 months of age with an osteopetrotic phenotype [48] and the defect was attributed to defective osteoclast function [55−59]. We therefore cultured mature osteoclasts harvested from Sam68^+/+^ and Sam68^−/−^ mice ex vivo on dentin slices to quantify their resorptive capacity. The fact that Sam68^−/−^ osteoclasts looked and acted like Sam68^+/+^ osteoclasts ex vivo and in vivo made it unlikely that this was the primary source of the difference in bone metabolism in 12-month-old Sam68^−/−^ mice. The fact that the CTX levels were lower in young and old Sam68^−/−^ mice suggested that there is reduced bone resorption compared with wild-type littermate controls. However, this reduction in bone resorption occurred with normal osteoclast activity, as assessed by in vitro culturing. These observations are consistent with the Sam68^−/−^ mice having a youth-like bone phenotype. However, it is still possible, that a mild impairment in Sam68^−/−^ mice osteoclast function may manifest itself later in life in overall accumulation of bone and this will require further detailed studies.

Ex vivo differentiation of primary bone marrow stromal cells, harvested from Sam68^+/+^ and Sam68^−/−^ mice, down the osteoblast lineage revealed an osteogenic advantage in the cultures of cells derived from the Sam68^−/−^ mice. It will be important to demonstrate that a similar effect is observed using primary bone marrow stromal cells from aged Sam68^−/−^ mice. Similar findings were observed when embryonic mesenchymal multipotential progenitor cells C3H10T1/2 treated with Sam68 shRNA, were differentiated into osteoblasts with BMP2. Our findings that aged Sam68^−/−^ mice do not develop fatty bone marrow and that MEFs derived from Sam68 ^−/−^ mice have impaired adipocyte differentiation, indicate that Sam68 regulates both adipocyte and osteoblast differentiation. These findings identify Sam68 as the first RNA binding protein to regulate mesenchymal cell differentiation and the challenge will be to identify the specific RNA targets that it regulates during this process. The fact that osteoblast function is altered in Src^−/−^ mice [60,61] raises the possibility that preservation of bone mass in the Sam68^−/−^ mice could be linked with and regulated by Src.

The pathway by which leptin regulates bone resorption was identified to involve the sympathetic nervous system relaying to the osteoblasts via the β-adrenergic pathway leading to the release of growth factors including RANKL that causes the osteoclasts to thrive [8,18–20]. The lowering of leptin levels in aged Sam68^−/−^ mice is consistent with these mice having a high bone mass compared with their aged littermates. These data would suggest that the leptin-sympathetic pathway is unaltered in Sam68^−/−^ mice and that the lowering of leptin may explain the lower levels of CTX in the serum of Sam68^−/−^ mice. These findings suggest that Sam68 may be regulating bone metabolism at two different levels: (1) the absence of Sam68 results in lower leptin levels that may reduce bone resorption via the sympathetic nervous system and (2) the absence of Sam68 favors osteoblast, rather than adipocyte, differentiation.

In conclusion, our data define a physiologic role for Sam68 in bone metabolism and bone marrow mesenchymal stem cell differentiation. The bone phenotype observed in Sam68^−/−^ mice imply that inhibitors of Sam68 could prevent age-related bone loss. Furthermore, the results also suggest that Sam68 expression levels, hypomorphism, and mutations in humans may influence susceptibility to marrow adipocyte accumulation and osteoporosis. Our findings also identify a new animal model to study aging bone loss.

## Materials and Methods

### Histologic, immunohistochemical, and histomorphometric analyses.

All analyses were performed essentially as described previously [62,63]. Briefly, embryonic mice were removed from timed pregnant dams at E14.5 and E16.5 and fixed intact for 36 h in 4% paraformaldehyde, rinsed thoroughly in PBS, and processed for paraffin embedding. Serial 4-μm sections were cut on a modified Leica RM 2155 rotary microtome (Leica Microsystems, Richmond Hill, Ontario, Canada), stained with a 1:600 dilution of the AD1 anti-Sam68 antibody [46] and counterstained with either methyl green or hematoxylin.

Adult mice were given an intraperitoneal injection of 30 mg/kg calcein at 7 days and 30 mg/kg tetracycline at 2 days prior to sacrifice to label actively mineralizing surfaces. After overnight fixation in 4% paraformaldehyde and rinsing in PBS, the left femur and tibia were embedded in polymethylmethacrylate (MMA) or a mixture of 50% MMA and 50% glycolmethacrylate (GMA). Serial 4- to 6-μm sections of MMA-embedded tissues were left unstained or stained with von Kossa and toluidine blue or with toluidine blue alone, while 4-μm MMA-GMA sections were stained for TRAP and ALP activity. Images were captured using a Leica DMR microscope (Leica Microsystems) equipped with a Retiga 1300 camera (Qimaging, Burnaby, British Columbia, Canada) and the primary histomorphometric data obtained using Bioquant Nova Prime image analysis software (Bioquant Image Analysis Corp, Nashville, Tennessee, United States). Nomenclature and abbreviations conform to those recommended by the American Society for Bone and Mineral Research [50].

### Generation of mice with targeted disruption of the *sam68* gene.

A λ bacteriophage clone encompassing Sam68 exons 3 to 9 was isolated from a 129/SvJ genomic library using full-length Sam68 cDNA as a probe. XbaI-digested Sam68 genomic DNA fragments of 4 kb (encompassing exon 4 and part of exon 5) and 3kb (spanning part of exon 5 and exon 6) were subcloned in Bluescript SK resulting in pBS4 and pBS3, respectively. A DNA fragment was amplified from pBS4 with the following oligonucleotides (5′-AAT GTC TAG AAA CAA CTC ATA TAC AGA C-3′) and the universal primer. The XbaI-digested 1-kb DNA fragment was subcloned in the XbaI site of pPNT (a gift from Andrew Karaplis, McGill University, Montréal, Quebec, Canada). The 3-kb fragment from pBS3 was amplified by PCR with (5′-GGG ATG CGG CCG CTC TAG AAT TGT CCT ACT TGA ACG G-3') and (5′-CGG TGG CGG CCG CTG TCG ACC TGA GTA ACA TTT CTT A-3′) and subcloned in the NotI site of pPNT. The targeting vector pPNT-Sam68 replaces exon 4 and part of exon 5 with a neomycin-resistant gene cassette. An SalI site was introduced at the 3′ end of the 3-kb DNA fragment and was used to linearize the plasmid for electroporation into embryonic stem (ES) cells. Approximately 1,000 ES colonies were screened and two clones were identified that contained the Sam68 mutant allele, as determined by Southern blotting. Targeted ES cells were injected into 3.5-day-old BALB/c blastocysts and were transferred into CD-1 foster mothers, and animals classified as chimeras by coat color were mated with BALB/c mice. Germ line transmission was achieved and the mice were maintained in C57BL/6 background. The mice used for this study represent mice that were backcrossed in C57BL/6 between three and eight generations; in addition, we maintained the mice in the 129/SvJ strain and observed a similar phenotype.

### Genotyping and immunoblot analyses.

All mouse procedures were performed in accordance with McGill University guidelines, which are set by the Canadian Council on Animal Care. Genomic DNA was isolated from tail biopsies and analyzed by Southern blotting and genomic PCR analysis. The DNA fragment utilized as the probe for the Southern blotting analysis was amplified with the following two oligonucleotides (5′-AAG CCT TTA CTG GTT GTG T-3′) and (5′-CTT GAA ACG CAC CGT AGG CT-3′). The wild-type *sam68* allele was identified by genomic PCR using the following oligonucleotides 5′-AAA TCC TAA CCC TCC TCA GTC AG-3′ and 5′-GAT ATG ATG GAT GAT ATC TGT CAG-3′. The *sam68*-targeted allele was identified by genomic PCR using the following oligonucleotides 5′-CTT GGG TGG AGA GGC TAT TCG-3′ and 5′-GTC GGG CAT GCG CGC CTT GAG C-3′.

### Radiology and serum biochemistry on Sam68^+/+^ and Sam68^−/−^ mice.

Radiography, BMD, and micro-CT were performed essentially as described previously [63]. Mice were administered a lethal dose of anesthetic at the indicated times, exsanguinated, and imaged using a Faxitron MX20 equipped with an FPX-2 Imaging system (Dalsa Medoptics, Waterloo, Ontario, Canada). Body fat, BMC, and BMD were evaluated using a Lunar PixiMUS 1.46 (GE-Lunar, Madison, Wisconsin, United States). Morphometric parameters were determined on anesthetized mice at the time of sacrifice by direct measurement or from the Faxitron radiograph.

Micro-CT was performed on the left femur and fourth lumbar vertebra after removal of soft tissues and overnight fixation in 4% paraformaldehyde. The distal metaphysis was scanned with a Skyscan 1072 micro-CT instrument (Skyscan, Antwerp, Belgium). Image acquisition was performed at 100 kV and 98 μA, with a 0.9° rotation between frames. The two-dimensional images were used to generate three-dimensional reconstructions to obtain quantitative data with the 3D Creator software supplied with the instrument.

Serum biochemistry (ALP, Ca, PO_4_, Mg) was determined at the Rodent Diagnostics Lab (McGill University, Montréal, Quebec, Canada) using routine automated techniques. Commercial assays were used to determine serum levels of CTX (RatLaps ELISA, Nordic Bioscience), 17β estradiol (IBL Immuno-Biological, Hamburg, Germany), leptin (R & D Systems, Minneapolis, Minnesota, United States), and interleukin-6 (R & D Systems).

### Ex vivo assessment of osteoblast and osteoclast activity.

Bone marrow was flushed from the tibia and femora of juvenile 4-week-old Sam68^+/+^ and Sam68^−/−^ mice to obtain stromal cells that were maintained in differentiation medium for 6 or 18 days as described [63]. Cultures were stained in situ for ALP activity and with von Kossa stain to detect mineralized nodules. Total RNA was harvested from parallel cultures for RT-PCR analysis of osteoblast-related gene expression over time as described previously [63].

Osteoclasts were isolated from the crushed long bones of juvenile Sam68^+/+^ and Sam68^−/−^ mice and used for quantitative studies as described [64]. Briefly, cells were plated on glass coverslips or on dentin slices in 24-well cluster plates for assessment of cell number and pit number, respectively. Cover slips were immersed in 4% paraformaldehyde and stained for TRAP activity after 2 h. Dentin slices were left for 28 h before removing the cells with 1 M ammonium hydroxide and air drying before coating with sputter Au-Pd and examination with scanning electron microscopy (McGill Electron Microscopy Centre). Light microscope images were captured using a Leica DMR microscope equipped with a Retiga 1300 camera. Quantitative analyses were performed using Adobe Photoshop and the data presented as the mean ± SD of two or three independent experiments. Statistical comparisons were made using the Student's *t* test.

### Preparation of mouse embryonic fibroblast and induction of adipocyte differentiation.

MEFs were prepared from 14.5-day-old embryos. Only early-passage MEFs were used for the experimental studies, and induction of adipocyte differentiation was carried out according to the methods previously described [30]. Cultured cells were stained with Oil Red O as described [29].

### RNA preparation and RT-PCR to assess adipogenesis in Sam68^−/−^ MEFs.

Total cellular RNA was prepared by TRIzol reagent according to the manufacturer's protocol (Invitrogen, Carlsbad, California, United States). Total RNA (1 μg) was reverse-transcribed, and cDNA samples were subjected to PCR. RT-PCR was normalized by the transcriptional level of GAPDH. The following 5′ and 3′ primers were used to evaluate adipogenic differentiation: KLF5: 5'-AGA CAA GCT GAG ATG CTG C-3′, 5′-GGC AAA CCT CCA GTC GC-3′; PPARγ: 5′-GTG CGA TCA AAG TAG AAC CTG C-3′, 5′-CCT ATC ATA AAT AAG CTT CAA TCG-3′; β-Actin: 5′-TAG GCG GAC TGT TAC TGA GC-3′, 5′-AGC CTT CAT ACA TCA AGT TGG-3′; and Sam68: 5′- GTG GAG ACC CCA AAT ATG CCC A-3′ and 5′-AAA CTG CTC CTG ACA GAT ATC A-3′.

### Sam68 down-regulation using Sam68sh.

To generate an shRNA, 64-base duplex DNA nucleotides were purchased from Invitrogen. This fragment comprised 19 base residues specific to mouse Sam68 (GATCCCC **AAGATGACGAGGAGAATTA**TTCAAGAGA** TAATTCTCCTCGTCATCTT** TTTTTGGAAA). This fragment was cloned into pSUPER retro (OligoEngine, Seattle, Washington, United States). Transfections were performed using LipofectAMINE Plus (Invitrogen) with cloned DNA fragment or empty vector. At 48 h after transfection, populations were selected for 2.5 μg/ml puromycin and the knockdown observed by immunoblotting with anti-Sam68 (AD1) antibody.

### Osteogenic differentiation analysis of C3HT101/2 cells.

For osteogenic differentiation, C3HT101/2 (ATCC) cells were incubated in 48-well tissue culture plates at 1.25 × 10^4^ cells/cm^2^ . After 24-h incubation, the culture media were changed to fresh media containing 300 ng/ml BMP-2 (Sigma, St. Louis, Missouri, United States). Total cellular RNA was prepared as described above, and osteocalcin, β-actin, and GAPDH gene expression at indicated times after BMP-2 treatment was quantified by RT-PCR.

## Supporting Information

Dataset S1 Localization of Sam68 in Primary Mouse Osteoblasts and Osteoclasts (124 KB PPT)

Dataset S2 Gene Expression Profile of Cultured Stromal Cells at Days 6 and 18 of Culture Isolated from Sam68^+/+^ and Sam68^−/−^ Mice (204 KB PPT)

Dataset S3 Serum Biochemistry and Bone Biomarkers in 4- and 12-Month-Old Mice (46 KB DOC)

## Abbreviations

ALP: alkaline phosphatase
BMC: bone mineral content
BMD: bone mineral density
BMP-2: bone morphogenetic protein-2
CT: computed tomography
CTX: C-telopeptide
MEF: mouse embryo fibroblast
PPARγ: peroxisome proliferator–activated receptor γ
shRNA: short hairpin RNA
TRAP: tartrate-resistant acid phosphatase
