# Supplementary material for: Ablation of the Sam68 RNA Binding Protein Protects Mice from Age-Related Bone Loss
Source: PLoS Genet. 2005 Dec 16;1(6):e74. doi: 10.1371/journal.pgen.0010074 (PMC1315279; doi:10.1371/journal.pgen.0010074)
Supplement: Supplementary file 1 [file pgen.0010074.sd003.doc]

**Dataset S3. Serum biochemistry and bone biomarkers in 4 and 12 month old mice**

| **Parameter** | **Units** | **Sam68^+/+^** | **Sam68^+/+^** | **Sam68^-/-^** | **mo Sam68^-/-^** |
| --- | --- | --- | --- | --- | --- |
| **Age (number)** |  | **4 mo (7)** | **12 mo (7)** | **4 mo (7)** | **12 mo (5)** |
| **sCTX** | **pmol/L** | 14.30 ± 2.58 | 19.10 ± 5.46 | 18.31 ± 2.51 | 13.21 ± 3.09^b, d^ |
| **Leptin** | **ng/ ml** | 2.40 ± 0.84 | 4.83 ± 2.22 ^a^ | 3.08 ± 1.45 | 2.13 ± 0.49 ^a^ |
| **17β estradiol** | **pg/ml** | 14.49 ± 2.89 | 11.51 ± 2.03 | 17.13 ± 3.44 | 11.56 ± 1.49 ^a^ |
| **IL-6** | **pg/ml** | 6.43 ± 2.93 | 13.32 ± 8.42 | 4.73 ± 4.11 | 13.46 ± 5.24 ^b^ |
| **ALP** | **U/L** | 50.80 ± 26.96 | 70.20 ± 15.90 | 81.40 ± 5.86 | 61.00 ± 23.66 |
| **Ca** | **mmol/L** | 2.37 ± 0.12 | 2.39 ± 0.03 | 2.52 ± 0.14 | 2.51 ± 0.12 |
| **PO_4_** | **mmol/L** | 2.41 ± 0.58 | 2.12 ± 0.40 | 2.41 ± 0.60 | 1.98 ± 0.35 |
| **Mg** | **mmol/L** | 1.16 ± 0.22 | 1.34 ± 0.29 | 1.21 ± 0.22 | 1.17 ± 0.12 |

sCTX: c-terminal telopeptide; 17β estradiol: estrogen; IL-6: interleukin 6; ALP: alkaline phosphatase; Ca: calcium; PO_4_: inorganic phosphate; Mg: magnesium.

*P* ^a^ < 0.01 and ^b^ <0.05 compared with 4 month mice of same genotype

*P* ^c^ < 0.01 and ^d^ <0.05 compared with wild type mice of the same age
